# New Insights into the Nephroprotective Potential of Lercanidipine

**DOI:** 10.3390/ijms241814048

**Published:** 2023-09-13

**Authors:** Joanna Hajdys, Piotr Fularski, Klaudia Leszto, Gabriela Majchrowicz, Magdalena Stabrawa, Ewelina Młynarska, Jacek Rysz, Beata Franczyk

**Affiliations:** Department of Nephrology, Hypertension and Family Medicine, Medical University of Lodz, Ul. Żeromskiego 113, 90-549 Lodz, Poland

**Keywords:** chronic kidney disease, lercanidipine, calcium channel blockers, dihydropyridines, renal protection

## Abstract

Kidneys are responsible for many crucial biological processes in the human body, including maintaining the water–electrolyte balance, pH, and blood pressure (BP), along with the elimination of toxins. Despite this, chronic kidney disease (CKD), which affects more and more people, is a disease that develops insidiously without causing any symptoms at first. The main purpose of this article is to summarize the existing literature on lercanidipine, with a particular focus on its nephroprotective properties. Lercanidipine is a third-generation dihydropyridine (DHP) blocker of calcium channels, and as such it possesses unique qualities such as high lipophilicity and high vascular selectivity. Furthermore, it acts by reversibly inhibiting L-type and T-type calcium channels responsible for exerting positive renal effects. It has been shown to reduce tissue inflammation and tubulointerstitial fibrosis, contributing to a decrease in proteinuria. Moreover, it exhibited antioxidative effects and increased expression of molecules responsible for repairing damaged tissues. It also decreased cell proliferation, preventing thickening of the vascular lumen. This article summarizes studies simultaneously comparing the effect of lercanidipine with other antihypertensive drugs. There is still a lack of studies on the medications used in patients with CKD, and an even greater lack of studies on those used in patients with concomitant hypertension. Therefore, further studies on lercanidipine and its potential in hypertensive patients with coexisting CKD are required.

## 1. Introduction

CKD is a medical term that describes a decline in kidney function or structure with health-related implications, lasting for at least 3 months, regardless of the cause. It may mean a decrease in glomerular filtration rate (GFR) below 60 mL/min/1.73 m^2^, or the existence of indicators of kidney damage, as well as both of them simultaneously. Kidney damage markers consist of at least one of the following: albuminuria, which is understood as an albumin excretion rate greater than or equal to 30 mg/24 h or an albumin-to-creatinine ratio greater than or equal to 3 mg/mmol (30 mg/g), abnormal urine sediment, disturbances in electrolytes and other irregularities caused by tubular disorders, abnormalities in histological examination, structural aberrations in imaging, or a prior history of renal transplantation [1,2].

This disease can be categorized into classifications abbreviated as CGA, with C standing for cause, G for GFR and A for albuminuria. There are six categories in CKD based on GFR and three categories based on the level of albuminuria. These categories, along with the values of individual stages, are presented in Table 1 and Table 2. When it comes down to the interpretation of the tables, starting from Table 1, G1 stands for normal or high GFR, G2 indicates a mild reduction in GFR, G3a means a mild to moderate reduction, G3b indicates a moderate to severe reduction, G4 represents a severe reduction in GFR, and G5 can be described as renal failure. Referring to the second table, the A1 stage stands for normal to mild elevation of albumin loss, A2 indicates moderate elevation and A3 indicates severe elevation [3].

Despite the fact that the kidneys regulate multiple physiological processes in the human body, including the water and electrolyte balance, pH, BP, the elimination of toxins and participation in the metabolism of vitamin D and the synthesis of selected hormones, there are no specific symptoms of CKD during its initial phases. For this reason, the disease often goes unrecognized at the beginning of its course [4]. However, the presence of the disease in certain patients can be suspected according to the fact that the main causes of CKD are diabetes mellitus (DM), high BP, persistent glomerulonephritis, persistent pyelonephritis, autoimmune diseases (ADs), polycystic kidney disease (PKD), and long-term abuse of non-steroidal anti-inflammatory drugs (NSAIDs), especially by patients suffering from chronic pain caused by rheumatological diseases [5,6]. Another group at risk of developing CKD includes those who are taking Chinese herbs, which may contain aristolochic acid, which can cause aristolochic acid nephropathy (AAN) [7]. What is more, this disease promotes hypertension via volume overload, sodium accumulation, increased sympathetic activity and changes in the hormonal mechanisms that control BP, which can lead to the development of resistant hypertension [8]. Furthermore, it is important to mention that people suffering from CKD are in danger of developing cardiovascular disease (CVD). Unfortunately, CKD causes a permanent decline in kidney function that can escalate to end-stage renal disease (ESRD). The ESRD requires the implementation of dialysis or renal transplantation; otherwise, this stage remains fatal, mainly due to CVD causes [9,10]. In this particular paper, our focus will be on a medication that exhibits significant renal protective effects, namely lercanidipine. Lercanidipine belongs to the DHP-CCBs, with an ability to lower BP due to the direct dilatation of both the efferent and the afferent glomerular arteries, while maintaining the intraglomerular capillary pressure at the same level [11]. Furthermore, by elevating the bioavailability of endothelial nitric oxide (NO), it shows anti-atherogenic traits. Additionally, it presents anti-inflammatory and antioxidant properties, as well as protecting the kidneys from damage induced by angiotensin II. Because of these and other positive effects, lercanidipine seems to be especially worth implementing in patients suffering from CKD [12].

## 2. Lercanidipine

### 2.1. Characterization of the Drug

Lercanidipine is a 1,4-DHP L- and T-type CCB [13]. It acts by preventing calcium intake into vascular and smooth muscle cells, which induces muscle relaxation and vasodilation and consequently causes a reduction in peripheral vascular resistance and a lowering of the BP. Lercanidipine is a member of the third generation of the DHP-CCBs, and as such it presents a long-lasting effect as well as high vascular selectivity [12,14]. Lercanidipine possesses two ester groups in positions 3 and 5 of the DHP ring, and thus it exists in the form of two different enantiomers—(S)-lercanidipine and (R)-lercanidipine. Studies have revealed that the (S)-enantiomer is the more potent one [15,16,17,18].

One of the most significant characteristics of lercanidipine is its high lipophilicity, a consequence of the drug’s phenylalkylamine side chain at the 3 position of the DHP ring, which guarantees the drug’s molecule easier penetration to its destination, as well as a higher concentration in the phospholipid membranes [14,15,16].

### 2.2. Pharmacology of Lercanidipine

After oral intake, the drug is largely absorbed from the gastrointestinal tract and goes through extended first-pass metabolism in the liver. The absorption of lercanidipine is increased in the presence of food, and hence it is recommended to administer the drug before meals [19,20,21]. Within the blood, lercanidipine extensively binds to plasma proteins—over 98% [20,22,23].

Lercanidipine, similarly to other DHP-CCBs, is metabolized in the liver by the isoform CYP3A4 of the cytochrome P450 (CYP) which, as it is important to remark, is involved in metabolizing up to 30% of all prescribed drugs. Therefore, regular use of other medication simultaneously with lercanidipine can possibly lead to adverse drug interactions [19,24,25,26,27].

According to the studies, the pharmacokinetic profile of lercanidipine is hardly altered in populations of patients like the elderly or those suffering from diseases such as cirrhosis or mild to moderate renal dysfunction. Thus, the administered dosage of lercanidipine for these patients does not need to be adjusted. However, in the instances of severe renal impairment when the estimated glomerular rate (eGFR) is lower than 30 mL/min/m^2^, the dosage needs to be reduced to avoid reaching high plasma concentrations [12,19,20,21,28,29,30].

The plasma half-life of lercanidipine is quite short—around 8 to 10 h. However, this does not correspond with the actual activity of the drug, which is significantly longer. The long-lasting antihypertensive effect of lercanidipine as well as its gradual onset of action can be explained by its high lipophilic qualities, which allow for the drug to be stored in the hydrophobic component of the cell. Therefore, lercanidipine can be administered once a day and allows for 24 h of BP control [20,22,23,28].

### 2.3. Mechanism of Action

Possibly the most important mechanism of action of lercanidipine is its ability to reversibly block high-voltage dependent L-type calcium channels that are expressed in cells of the cardiac tissue, skeletal muscles and all excitable cells [19,31,32,33]. The inhibition of these channels, that are present in the cardiovascular system (CVS), allows for a reduction in the peripheral vascular resistance via relaxation of the arterial smooth muscles and peripheral as well as coronary vasodilation [23,33,34,35]. Therefore, lercanidipine exerts antihypertensive and anti-ischemic effects [33]. Remarkably, the lowering of the BP caused by lercanidipine is not associated with unwanted reflex tachycardia, due to the drug’s slow onset of action, or other adverse signs of sympathetic activation [23,36,37,38,39].

Furthermore, lercanidipine is able to inhibit T-type low-voltage calcium channels which are present in various organs, such as the heart or the kidneys, where they are an important molecular target. Studies have shown that CCBs that inhibit both L- and T-type calcium channels, such as lercanidipine, improve kidney function, reduce glomerular hypertension and proteinuria and exert an overall positive effect on glomerular morphology [13,40,41].

Lercanidipine has been shown to possess beneficial anti-atherogenic qualities. Preclinical studies have concluded that it decreases atherosclerotic lesions in hypercholesterolemic rabbits and additionally inhibits proliferation and migration of the arterial smooth muscle cells [17,22,42].

In addition, there have been reports suggesting that lercanidipine may be involved in end-organ protection. Studies have found that the drug reduces left ventricular hypertrophy [19,43,44]. Moreover, preclinical data have indicated that the administration of lercanidipine decreases the incidence of cerebral stroke (CS) [19,45].

### 2.4. Indication for Administration

The principal indication for lercanidipine is hypertension. Several studies have shown that lercanidipine in monotherapy is an effective form of treatment of hypertension [22,46,47,48,49,50,51,52]. It has been concluded that lercanidipine in a dosage of 5 to 20 mg taken once a day is able to reduce BP in patients suffering from mild to moderate hypertension [19,53,54,55,56]. A non-blind study found that monotherapy with lercanidipine at a dose of 20–40 mg per day successfully decreases BP in patients with severe essential hypertension [19,57]. An unpublished study also showed lercanidipine at a dose of 10–30 mg per day to be an effective form of treatment for resistant hypertension unresponsive to other drug classes [19,58]. Moreover, lercanidipine is successful in treating elderly patients with mild to moderate essential hypertension as well as isolated systolic hypertension [19,37,53].

However, the new European Society of Hypertension/European Society of Cardiology guidelines from 2018 recommend administering a combination of two drugs to control a hypertensive patient, as multitherapy was concluded to be more beneficial [59,60]. Therefore, the initial treatment in most cases should consist of a renin––angiotensin system blocker and a CCB or a diuretic. The administration of CCBs, such as lercanidipine, in this combination is especially advantageous in patients with diabetes, coronary artery disease (CAD), cerebrovascular disease (recommendations of grade IA) [59,60].

Furthermore, studies have shown that combining lercanidipine with any type of drug allows for a more significant reduction in BP than that exerted by lercanidipine alone [49,59,61].

### 2.5. Absorption

Lercanidipine is a drug that is slowly absorbed from the gastrointestinal tract after oral administration. The maximum drug concentration (Cmax) in the plasma is reached within 1.5–3 h [11,12,62]. Lercanidipine is extensively metabolized during the first hepatic flux, with an absolute bioavailability of 10% in the fed patient [63,64,65]. Lercanidipine should be taken before meals as its absolute oral bioavailability is increased by the presence of a high-fat meal or the addition of surfactants. One study developed lercanidipine–hydroxypropylmethyl cellulose (HPMC) nanoparticles to which d-tocopheryl polyethylene glycol 1000 succinate (TPGS) was added, and this combination was shown to have 2.47 times higher oral bioavailability than the raw material alone [66].

### 2.6. Adverse Effects

This review summarizes the possible adverse effects (AEs) of lercanidipine. The supervention of AEs in patients taking lercanidipine has been associated with vasodilation in the early stages of treatment [23,67]. The incidence of AEs and the safety of lercanidipine have been studied in 14 double-blind, placebo-controlled clinical trials. In total, 1850 people with hypertension or stable angina pectoris (SAP) participated in all studies. Patients were randomized and most of them were treated with lercanidipine at doses of 10–20 mg for up to 129 days [68]. The proportion of patients reporting adverse reactions following treatment with lercanidipine was 26.8%, with the most commonly reported adverse reactions being headache (5.6%), edema (2.4%), tachycardia (2.1%), flushing (2.0%), palpitations (1.7%), rhinitis (1.3%) and hypokalaemia (1.2%). In turn, patients receiving placebo accounted for 20.3% of all respondents, and the most frequently reported adverse reactions were headache (3.8%), hypokalaemia (1.3%) and hyperuricemia (1.1%). The vast majority of patients experienced mild or moderate AEs; however, more importantly, most of these were considered unrelated to lercanidipine. A randomized, open-label, controlled study was also conducted in a group of 104 hypertensive patients diagnosed with ischemic stroke, which showed that the rate of AEs that occurred after the use of lercanidipine was negligible. One of the AEs was facial flushing (*n* = 3; 5.7%), and the other reported adverse reaction was ankle edema, which occurred in only two patients (3.8%) [69]. In another multicenter, prospective, non-comparative, open-label study, the study population consisted of 3175 patients treated with lercanidipine for 6 months. Depending on the cardiovascular risk, patients were divided into four groups: low, medium, high and very high cardiovascular risk. One of the endpoints of this study was the presentation of the side effects of this drug. Adverse reactions after the use of lercanidipine occurred in 11.5% of the study population, the most common of which was edema (5.1%), followed by headache (3.3%), flushing (2.5%), and asthenia (1%). Differences in the incidence of AEs by cardiovascular risk group were not significantly significant [52].

### 2.7. Contraindications

Hepatic or renal insufficiency (creatinine clearance <10 mL/min) is a contraindication to the use of lercanidipine therapy. Lercanidipine is also contraindicated in pregnant and lactating women and in women of childbearing potential not using effective contraception. Left ventricular outflow tract obstruction (LVOTO), untreated congestive heart failure, unstable angina pectoris and within 1 month of myocardial infarction are clinical conditions in which the use of lercanidipine is contraindicated [20,52]. The combination of lercanidipine with strong CYP3A4 inhibitors or CsA is also contraindicated [70]. CsA is an immunosuppressive drug, and its combination with lercanidipine increases the concentration of CsA in the blood serum, which may contribute to the occurrence of various side effects [71]. Elderly patients or patients with mild to moderate renal or hepatic impairment should be treated with special caution when incorporating lercanidipine into their treatment [19]. Lercanidipine is also not recommended for people under 18 years of age [20].

### 2.8. Interactions

Lercanidipine is metabolized by CYP, more specifically its CYP3A4 isoform [24]. The CYP3A4 isoform is mainly involved in the metabolism of drugs in the liver and intestines [26,72]. CYP3A4 is the most abundant P450 in the human liver, accounting for 30% of the total CYP content. It is also found in the prostate, breast, intestine, colon, small intestine, and brain [72].

Ketoconazole is an example of a potent CYP3A4 inhibitor. As we already know, lercanidipine is metabolized by this enzyme, and therefore we can expect that the plasma concentrations of lercanidipine may be altered if the two drugs interact. Studies have shown that co-administration of these substances resulted in an 8-fold increase in lercanidipine Cmax and increased the area under the curve (AUC) of lercanidipine by about 15-fold [20,22,24,70,73]. Cyclosporine (Cyclosporin A, CsA) is a drug with strong immunosuppressive properties, widely used in transplantology, but its use poses a high risk of drug interactions [74,75]. As a result of the concomitant use of CsA (CYP3A4 substrate and inhibitor), the bioavailability of lercanidipine increased three-fold, while the plasma concentration of ciclosporin increased by 21%. In patients taking CsA and lercanidipine concomitantly, it is important to monitor CsA concentrations due to the possibility of side effects [24,71,76,77]. The oral bioavailability of lercanidipine will also be enhanced by more specific CYP3A4 inhibitors, for example grapefruit juice. As a result of the combination of these two drugs, a stronger antihypertensive effect will be observed compared to monotherapy with lercanidipine 10 mg [24,78].

Midazolam is a common substrate for CYP3A4 and metoprolol for CYP2D6. Studies have shown that there are no significant pharmacokinetic interactions when midazolam is co-administered with lercanidipine or metoprolol with lercanidipine. The bioavailability of both midazolam and metoprolol was unaffected by lercanidipine administration, but dose adjustment may be necessary [11,24,79,80]. Concomitant use of β-methyldigoxin and lercanidipine in doses of 10–20 mg also did not cause clinically significant interactions; however, monitoring of patients who use a combination of these drugs is recommended due to the possibility of digoxin toxicity [20,24].

Cimetidine is one of the inhibitors of the CYP3A4 enzyme; however, when co-administered with lercanidipine, the main pharmacokinetic parameters and plasma concentrations of lercanidipine were not significantly altered. Due to this fact, there is no need to adjust the dose of the drugs when they are used concomitantly [21,24]. Simvastatin belongs to the group of statins, and it is a prodrug (inactive molecule) that is then converted into beta-hydroxy acid (active molecule), metabolized mainly by CYP3A4 [81]. In one study, co-administration of lercanidipine at a dose of 20 mg with simvastatin at a dose of 40 mg resulted in a 56% increase in the bioavailability of simvastatin and a 28% increase in its active β-hydroxy acid metabolite. The bioavailability of lercanidipine was not changed. Accordingly, it is recommended that the dosing of lercanidipine and simvastatin be separated in time (lercanidipine taken in the morning and simvastatin in the evening) [82]. Another study reported an interaction between lercanidipine and one of the antipsychotics, haloperidol, with this combination leading to hypotension. The highly probable cause of this interaction was the inhibition of lercanidipine metabolism by haloperidol (CYP 3A4 isoenzyme inhibitor) [83]. Concomitant use of fluoxetine and lercanidipine may lead to hypotension. The source of this interaction may be competition for binding with the CYP3A4 isoenzyme, which metabolizes both fluoxetine and lercanidipine, resulting in increased plasma concentrations of lercanidipine and AE. Paroxetine and sertraline share the same mechanism of interaction with lercanidipine as fluoxetine, but the side effects of these interactions are different. In the case of the combination of paroxetine and lercanidipine, the result was polyuria and increased levels of transaminases, while the combination of sertraline and lercanidipine caused myalgia and polyuria [84].

## 3. Nephroprotective Effect of Lercanidipine

Lercanidipine, as a compound of a new generation of CCBs, exhibits pleiotropic actions that are not due to the class effect of these agents but rather them having additional qualities [85,86,87,88]. They not only influence the reduction in BP, but also exert positive effects on renal parameters, thus limiting their progressive damage [12,59].

In preclinical studies on hypertensive rats, it was observed that lercanidipine dilated both afferent and efferent arterioles in the kidneys therefore reducing intraglomerular pressure or maintaining it at the same level [11,40,41,88,89,90]. The mechanism was associated with the blockade of both L-type calcium channels, which is typical of most CCBs, but also T-type calcium channels [59,91]. T-type calcium channels are predominantly specific to the efferent arterioles, although they also occur in the afferent arterioles [40,92]. The combined blockade of L and T channels has been shown to have positive effects on renal function in CKD by reducing proteinuria and enhancing renal viability [86,93,94,95,96,97]. The simultaneous effect on L-type and T-type calcium channels results not only in a decline in BP, but also in a lowering of glomerular filtration fraction, prompting the new generation of drugs to exert similar nephroprotective effects as drugs blocking the renin–angiotensin axis (RAA) [88,98].

In preclinical studies, lercanidipine prevented thickening of the vascular middle membrane and vascular neointima of the walls of small arteries and arterioles, including renal ones, through suppression of cell proliferation and thereby preventing the narrowing of their lumens [99,100,101]. Furthermore, it reduced tissue inflammation and tubulointerstitial fibrosis, associated with the preservation of renal function and reduced albuminuria in double-transgenic rats [41,90].

Lercanidipine exhibits antioxidant effects, as evidenced in preclinical and clinical studies [102,103,104]. It causes a reduced production of free radicals by inhibiting enzymes that are their main intracellular source, including NADPH oxidase (NOX), xanthine oxidase (XO) and cyclooxygenase (COX) [86,105,106]. In addition, it decreases oxidative stress markers such as plasma lipoperoxidase, isoprostanes (IsoPs), malondialdehyde (MDA), metalloproteinase-9 (MMP-9), asymmetric dimethylarginine (ADMA), myeloperoxidase (MPO), leukocyte-derived vascular NO oxidase, and endogenous nitric oxide synthase (NOS) inhibitors [85,100,104,107]. Through the reduction in the levels of cellular reactive oxygen species, it also inhibited cholesterol accumulation [42,108].

While inhibiting NO oxidase and decreasing NOS inhibitors immunoreactivity, lercanidipine leads to an enhancement of NO bioavailability in the blood vessels and glomeruli, which along with its anti-inflammatory effect may be responsible for reducing monocyte infiltration, extracellular matrix formation and fibrosis in renal vessels [100].

It was observed that the increase in the number of mesangial cells disrupts the transport of macromolecules within the mesangium and can induce quantitative or qualitative changes in mesangial matrix proteins, hence contributing to progressive glomerular sclerosis [109,110,111,112]. Lercanidipine counteracts these mechanisms by restraining mesangial cell proliferation [100,113] due to the inhibition of activator protein-1 (AP-1) [114] and by inhibiting the cell cycle transition from the G1 to the S phase [115]. Moreover, it modulates the transcription of interleukin-1 beta (IL-1β) and granulocyte/monocyte colony-stimulating factor genes, which are induced in mesangial cells by platelet-derived growth factors (PDGFs) [116,117].

According to the studies, lercanidipine has been observed to induce diminished activation of intracellular protein kinases in particular protein kinase C (PKC) isoforms and activation of the ADMA-metabolizing enzyme dimethylarginine dimethylaminohydrolase, while also enhancing NO concentration, probably due to reduced cellular calcium concentration [100,118,119,120]. Reduced PKC activity was also associated with decreased permeability of albumin by endothelial cells [121]. In patients, lercanidipine has been shown to reduce the blood levels of C-reactive protein, lipoprotein A, E-selectin and P-selectin [122,123]. In experimental conditions, as well as later in clinical studies, a reduction in the expression of intercellular adhesion molecules (ICAMs) involved in vascular and tissue damage was noted [123]. It was additionally observed that lercanidipine increased the expression of fibronectin, thereby contributing to the restoration of damaged tissues [100].

A new class of CCBs, including lercanidipine, has been shown to reduce norepinephrine secretion [40,124,125] as well as inhibit the renal action of endothelin [29,105], which further enhanced the vasoconstrictive properties of norepinephrine [126]. The attenuated effects of the above compounds suppressed renal vasoconstriction, therefore preventing further endothelial dysfunction [38,127]. The most relevant nephroprotective mechanisms of lercanidipine are summarized in the table below (Table 3).

## 4. Lercanidipine as Hypertensive Medicament and Its Comparison with Other Antihypertensive Drugs

### 4.1. Efficacy of Lercanidipine

Clinical trials have shown that lercanidipine is an effective and well-tolerated drug for hypertension, both in newly diagnosed patients and in those whose previous treatment has not been successful [49,50,52,128]. In a study by Burnier et al. [128], lercanidipine showed comparable reductions in BP in never-treated hypertensive patients and patients who were transferred to lercanidipine because they either had inadequately controlled BP or had AEs from previous treatment. Similar observations were noted in the ELYPSE study [49]. Furthermore, only 6.5% of patients reported experiencing AEs during the study [49].

Lercanidipine can be used safely in the elderly and in patients with metabolic syndrome [37,50,128]. In the Viviani study [129], the use of lercanidipine in diabetic patients showed not only a significant decrease in patients’ BP, but also a meaningful decrease in blood glucose, glycated hemoglobin A1 (HbA1) and fructosamine. Moreover, in the Acanfora et al. study [130], lercanidipine was effective in reducing the signs and symptoms of ischemia in patients with stable angina, and also did not cause sympathetic activation. The LAURA study [52] found that the greater the cardiovascular risk, the greater the improvement in BP with lercanidipine without notably altering its tolerability.

### 4.2. Comparison of DHP CCBs and Non-DHP CCBs

Taking into consideration the extent and mechanism of nephroprotection, research has observed differences between the effects of two subclasses of calcium antagonists: DHP CCBs (which includes lercanidipine) and non-DHP CCBs [131,132]. Even preclinical studies have proven that non-DHPs can inhibit the increase in protein levels in the urine and slow down the production of the mesangial matrix and the process of glomerular scarring [131]. Meta-analysis assessing the effects of the subclasses on BP parameters and the level of proteinuria among patients with arterial hypertension has clearly shown that non-DHP CCBs contribute to a greater decrease in proteinuria level with similar results on the hypotensive effect [132].

### 4.3. Comparison of Lercanidipine with Other DHP-CCBs

The protective effect of hypotensive drugs on kidneys has two main mechanisms: the lowering of BP and intrarenal processes [133]. According to statistical analysis, the first one, namely the antihypertensive effect of lercanidipine, does not significantly differ from other DHP-CCBs [86,134]. However, lercanidipine, as a new generation of DHP-CCBs, displays a higher nephroprotective potential on the intracellular level in comparison with conventional DHP-CCBs.

The analyses evaluating the risk of adverse reactions in new and classical DHP-CCB therapies suggest that the use of lercanidipine is associated with reducing the exacerbation [86] and frequency [134] of AE, mainly peripheral edema [86,134], and consequently also lowering the risk of dechallenging caused by this AE [131]. According to the analysis led by Makani et al., the application of lipophilic calcium antagonists decreases the risk of edemas by 57% [135]. Other trials have also found a significantly lower incidence of AEs after lercanidipine than after other CCBs [46,50,51].

The ELderly and LErcanidipine (ELLE) study designed by Antonio Cherubini et al. [46] has shown that lercanidipine exhibits the best antihypertensive effect compared to lacidipine and nifedipine. Moreover, lercanidipine had the lowest incidence of ADRs, which proves lercanidipine to be the most effective and safe drug among these three [46]. Furthermore, the TOLERANCE study designed by Barrios et al. [51], whose aim was to compare the tolerability of high doses of lercanidipine with high doses of amlodipine and nifedipine, with the main variable being adverse effects related to vasodilation, found that the incidence of these ADRs was significantly lower in the lercanidipine group. Therefore, administration of lercanidipine seems to be an appropriate option for patients in need of high doses of medication. The study by Leonetti et al. [48], which compared the tolerability of long-term treatment with lercanidipine versus other CCBs in elderly hypertensive patients, found lercanidipine to have a better tolerance profile than amlodipine and a similar one to lacidipine while maintaining an equivalent antihypertensive effect, proving it to be a suitable option for patients of older age. Moreover, while previous studies have shown that the group of CCBs can play a role in stroke prevention [136,137,138], the retrospective 6-year study by Cheng et al. [136] concluded that lercanidipine was significantly more effective in reducing the risk of stroke than nifedipine.

### 4.4. Comparison of Lercanidipine with Other Antihypertensive Drugs

Angiotensin-converting enzyme inhibitors (ACE-I) are another well-known group of medications that are commonly used in treating hypertension, congestive heart failure and nephropathies [139,140]. A study by Derosa et al. [139] compared the effects of lercanidipine and enalapril in monotherapy with those of enalapril and lercanidipine in a fixed combination and found that the combination of both drugs was more effective in reducing BP than the single monotherapies. The same results were observed in other studies, such as Mancia et al. [141], Mancia et al. [142] and Puig et al. [143]. A similar study by Yang et al. [144], comparing the efficacy of lercanidipine and perindopril in monotherapy and in fixed combination, also found the combination of these drugs to be the most tolerable and the most effective in lowering BP. However, when comparing the efficiency of both monotherapies, lercanidipine alone seemed to exhibit a more significant reduction in BP than perindopril [144].

Angiotensin II type 1 receptor antagonists (ARBs), drugs with similar mechanisms of action to ACE-I, with both of them inhibiting the renin–angiotensin–aldosterone system (RAAS), are commonly used in the treatment of hypertension as well [59,145]. A study designed by James et al. [146], which compared the efficacy and tolerability of lercanidipine and losartan, found that lercanidipine in monotherapy was more effective in normalizing BP than losartan alone. However, a study by Na et al. [147], which compared the efficacy and safety of lercanidipine in monotherapy versus lercanidipine and valsartan combination, observed that the drug combination exhibited the best antihypertensive effect.

These findings are consistent with the European Society of Hypertension/European Society of Cardiology (ESH/ESC) guidelines, which recommend multitherapy as a treatment for hypertension [59,60]. The evidence has shown that a multitherapy regimen consisting of CCB, such as lercanidipine, and a RAAS inhibitor is effective in reducing BP because of the complementary mechanisms of action of these drugs [61,147,148,149,150,151]. Moreover, it is suggested that combining lercanidipine with an ACE-I or ARB can lower triglyceride and glucose levels [61,147].

Administering multitherapy as a treatment for hypertension can have many benefits for the overall health of the patients [59]. A study by Ghiadoni et al. [152] has shown that the multitherapy of lercanidipine and enalapril reduced the augmentation index more significantly compared to the multitherapy of hydrochlorothiazide and enalapril. This suggests that this combination might have an additive role for cardiovascular protection. Moreover, a study by Tsioufis et al. [153] has found the enalapril and lercanidipine combination to reduce hypertension-related target organ damage and improve kidney functions the most compared to combinations of enalapril with amlodipine and enalapril with hydrochlorothiazide. The RED LEVEL study by Robles et al. [154], which compared combination therapy of enalapril plus lercanidipine with enalapril plus amlodipine, found that, while there was no significant difference in BP control between the two groups, the lercanidipine plus enalapril combination had a superior anti-albuminuric effect. Similar results were shown in a study by Fici et al. [155], which found that, among treatments with different combinations of RAAS blockers and CCBs, multitherapy of lercanidipine with enalapril had the highest rate of albuminuria reduction. This evidence might favor the administration of lercanidipine combined with RAAS inhibitors to control BP and the albuminuria rate in diabetic patients with hypertension [154,155]. From all of the studies mentioned above, the ones most significant to our research are summarized in Table 4.

In clinical practice, patients at high cardiovascular risk who also suffer from kidney disease remain a challenge. Such patients require an integrated approach and well-selected pharmacotherapy. One of the possible combinations used by clinicians in such cases is the combination of SGLT-2 inhibitors with antihypertensive drugs, including DHP-CCB. SGLT-2 inhibitors selectively inhibit sodium-glucose cotransporter 2, thereby blocking the reabsorption of filtered glucose, resulting in glycosuria [156]. These are drugs with cardio- and nephroprotective effects, which also prevent hyperkalemia and have a weak hypotensive effect [157,158]. They are extremely useful in patients with type 2 diabetes with additional cardiovascular or nephrological burden [157,159].

It should be noted that DHP-CCB may interact with iSGLT-2 in renal hemodynamic function [160]. A post hoc analysis of the EMPA-REG OUTCOME study was performed to check whether there are interactions between iSGLT-2 and, among others, CCBs. Although the results of this study did not show any significant interactions between these drugs in terms of effectiveness and safety, it should be noted that the study had several limitations (including potential bias, small subgroups) that could affect the results [160,161].

To summarize, despite the limited number of observations in this area, we can conclude that DHP-CCBs can probably modify the hemodynamic effect of iSGLT-2, but to a very small extent [160].

## 5. Conclusions

Kidneys are important organs that are responsible for many biological processes around the whole body, including BP regulation, water and electrolyte balance maintenance, pH control, the elimination of toxins and more. Nowadays, along with the increased frequency of lifestyle-related diseases like DM or hypertension, the frequency of CKD occurrence is also rising. The absence of specific symptoms at the beginning can later result in the presence of severe disorders like resistant hypertension, which contributes to the progression of the disease. To counteract this, it is worth implementing appropriate nephroprotection measures, such as using lercanidipine.

Lercanidipine is a third-generation DHP-CCB, and as such it possesses unique qualities—high lipophilicity, which guarantees a long-lasting effect of the drug, and high vascular selectivity. Lercanidipine acts by reversibly inhibiting L-type and T-type calcium channels, and thus it reduces peripheral vascular resistance and exerts antihypertensive and anti-ischemic effects. Lercanidipine exerts positive effects on renal parameters. It has been shown to reduce tissue inflammation and tubulointerstitial fibrosis, contributing to a decrease in proteinuria. Moreover, it exhibited antioxidative effects and increased expression of molecules responsible for repairing damaged tissues.

Several studies have concluded that lercanidipine is an extremely effective drug for hypertension that can safely be used in special patient populations such as the elderly, diabetics and patients with metabolic syndrome. Compared to other CCBs, lercanidipine therapy has been shown to allow for a better BP control, significant improvements in eGFR and a great tolerability profile. Moreover, several studies have found lercanidipine in monotherapy to be an efficient antihypertensive agent, although it has been found that combining it with other antihypertensive medications such as RAAS inhibitors in multitherapy allowed for many beneficial effects including a reduction in albuminuria, and a possible reduction in cardiovascular risk.

However, it is important to note that lercanidipine causes AEs associated with vasodilation at the start of treatment. The most commonly reported symptoms were edema, headache, flushing and tachycardia. Before initiating lercanidipine, contraindications should be noted, as well as the fact that it is metabolized by CYP3A4 and may interact with inducers or inhibitors of this enzyme.

## Figures and Tables

**Table 1:** Categories of CKD based on GFR [3].

**Category Based on GFR**	**Category**	**GFR (mL/min/1.73 m^2^)**
	G1	≥90
	G2	89–60
	G3a	59–45
	G3b	44–30
	G4	29–15
	G5	<15

**Table 2:** Categories of persistent albuminuria in CKD [3].

**Category Based on Albuminuria**	**Category**	**AER**	**ACR**
	A1	<30 mg/day	<3 mg/mmol <30 mg/g
	A2	30–300 mg/day	3–30 mg/mmol 30–300 mg/g
	A3	>300 mg/day	>30 mg/mmol >300 mg/g

Abbreviations: albumin excretion rate (AER); albumin–creatinine ratio (ACR).

**Table 3:** Nephroprotective mechanisms of lercanidipine along with their exerted influence.

Mechanisms of Kidney Protection	Exerted Influence
Affecting both L-type and T-type calcium channels	Reduction in BP while maintaining constant intraglomerular pressure
Suppression of cell proliferation in renal arterioles	Prevention of thickening of the vascular middle membrane and vascular neointima, prevention of lumen narrowing
Reduction in tissue inflammation and tubulointerstitial fibrosis	Decrease in albuminuria, preservation of renal function
Inhibition of free radicals producing enzymes	Exerting antioxidant effects
Decrease in oxidative stress markers	
Increase in NO bioavailability in blood vessels and glomeruli	Reduction in monocyte infiltration, extracellular matrix formation and fibrosis in renal vessels
Inhibition of cholesterol accumulation	Anti-atherosclerotic effect
Inhibition of AP-1 and the cell cycle transition from G1 to S phase of mesangial cells	Inhibition of mesangial cell proliferation
Modulation of transcription of IL-1β and granulocyte/monocyte colony stimulating factor genes in mesangial cells	
Reduction in PKC activity	Decrease in permeability of albumin by glomerular endothelial cells
Decrease in expression of intracellular adhesion molecules	Reduction in blood vessel and tissue damage
Increase in fibronectin expression	Restoration of damaged tissue
Renal endothelin inhibition	Decrease in the impact on norepinephrine activity
Reduction in norepinephrine secretion	Reduction in neurally induced vasoconstriction and prevention of further endothelial dysfunction

Abbreviations: AP-1 activator protein-1; PKC protein kinase C; IL-1β interleukin-1 beta.

**Table 4:** Lercanidipine and its efficacy in mono- and multitherapy.

Study	Type of Study	Duration	Patients (*n*)	Hypertensive Patient Category	Treatment	Remarks
Cherubini et al., 2003 [46]	Multicenter, double-blind, randomized, parallel group study	24 weeks	324 (aged 65 years or above)	Mild-to-moderate essential systolic and diastolic hypertension	Lercanidipine 5 mg vs. lacidipine 2 mg vs. nifedipine 30 mg	The mean BP difference was greater in the lercanidipine group compared to the lacidipine group and nifedipine group, respectively. The ADR rate was the lowest in the lercanidipine group.
Barrios et al., 2008 [51]	Observational, transversal, multicentre study	3.6 months	650 (aged ≥18 years)	Essential hypertension	Lercanidipine 20 mg vs. amlodipine10 mg vs. nifedipine GITS 60 mg	BP control was comparable between the groups. Adverse effects related to vasodilation were significantly more frequent in the amlodipine/nifedipine group compared to the lercanidipine group).
Leonetti et al., 2002 [48]	Multicenter, double-blind, parallel study	12 months	828 (aged ≥60 years)	Essential hypertension	Lercanidipine 10 mg vs. amlodipine 5 mg vs. lacidipine 2 mg	BP control was comparable between the groups. Edema-related adverse symptoms (such as lower limb swelling and heaviness) occurred more frequently with amlodipine compared to lercanidipine and lacidipine.
Cheng et al., 2017 [136]	Observational, retrospective cohort study	6 years	144,630 (aged 18 to 65 years)	Essential hypertension	Continuous therapy with lercanidipine vs. nifedipine vs. amlodipine vs. felodipine (the dosage was not mentioned in the study)	No difference in any endpoint was found between lercanidipine and other groups. Lercanidipine was shown to be significantly more successful vs. nifedipine in reducing the incidence of stroke.
Derosa et al., 2014 [139]	Multicenter, randomized, double-blind, clinical study	24 months	345 (aged <65 years)	Essential hypertension	Enalapril 20 mg vs. lercanidipine 10 mg vs. enalapril/lercanidipine 20/10 mg	Reduction in BP was greater in the enalapril/lercanidIpine group compared to the other groups. Lercanidipine improved lipoprotein(a) levels, while the combination of enalapril/lercanidipine improved it more than single therapies. Other biomarkers of cardiovascular risk were improved by all three therapies, but the greatest effect was observed in the enalapril/lercanidipine group.
Mancia et al., 2014 [141]	Randomized, double-blind, placebo-controlled, parallel group study	12 weeks	1039 (aged 18 to 75 years)	Moderate hypertension	Lercanidipine 10 or 20 mg vs. enalapril 10 or 20 mg vs. enalapril/lercanidipine 10/10 mg or 20/10 mg or 10/20 mg or 20/20 mg	DBP and SBP were reduced more significantly in the enalapril/lercanidipine 20/20 mg group compared to other combinations and monotherapy.
Mancia et al., 2016 [142]	Multicenter, randomized, double blind, parallel group study	12 weeks	854 (aged 18 to 75 years)	Grade 1 or 2 essential hypertension	Lercanidipine 10 or 20 mg vs. enalapril 10 or 20 mg vs. lercanidipine/enalapril 10/10 mg or 20/10 mg or 10/20 mg or 20/20 mg	Reduction in BP was more significant in the enalapril/lercanidipine combination compared to the other groups.
Puig et al., 2007 [143]	Randomized, double-blind, placebo-controlled, four-way crossover study	18 weeks	75 (aged 60 to 85 years)	Essential hypertension	Lercanidipine 10 mg vs. enalapril 20 mg vs. lercanidipine/enalapril 10/20 mg	Reduction in BP was significantly more effective in the enalapril/lercanidipine group compared to the other groups.
Yang et al., 2014 [144]	Monocentric, randomized, controlled study	12 weeks	180 (aged 18 to 70 years)	Mild essential hypertension	Perindopril/lercanidipine 2/5 mg vs. lercanidipine 10 mg vs. perindopril 4 mg	The normalization rate of BP was the highest in the perindopril/lercanidipine group compared to the other groups. The ADR rate was the lowest in the perindopril/lercanidipine group.
James et al., 2002 [146]	Randomized, double-blind, double- dummy, parallel group study	18–20 weeks	562 (aged 18–75 years)	Mild to moderate essential hypertension	Lercanidipine 10 mg vs. losartan 50 mg	The normalization rate of BP was higher in the lercanidipine group compared to the losartan group. Lercanidipine had a better dose response than losartan. The ADR rate was lower in the lercanidipine group compared to the losartan group.
Na et al., 2015 [147]	Randomized, double-blind, multi- center, parallel group, Phase III clinical trial	20 weeks	772 (aged 20 to 75 years)	Essential hypertension	Lercanidipine 10 mg vs. lercanidipine/valsartan 10/80 mg vs. lercanidipine/valsartan 10/160 mg	Combination therapy was more effective in treating hypertension compared to lercanidipine alone.
Ghiadoni et al., 2015 [152]	Prospective, randomized, open, with blinded end-points (PROBE), parallel-group study	28 weeks	118	Essential hypertension	Enalapril/lercanidipine 20/10 mg (up-titrated to 20/20 mg) vs. enalapril/hydrochlorothiazide 20/12,5 mg (up-titrated to 20/25 mg)	The combination therapy of enalapril/lercanidipine reduced the central augmentation index more significantly compared to the other group. Reduction in BP and arterial stiffness was similar among the groups.
Tsioufis et al., 2016 [153]	Randomized, blinded-endpoint trial	3 months	56	Grade 2 essential hypertension	Enalapril/lercanidipine 20/10 mg vs. enalapril/amlodipine 20/5 mg vs. enalapril/hydrochlorothiazide 20/12,5 mg	The combination of lercanidipine and enalapril improved the hypertension-related target organ damage the most significantly among all the groups. A significant decrease in the renal arterial restrictive index and the urinary albumin-to-creatinine ratio was noted in the enalapril/lercanidipine group.
Robles et al.,2016 [154]	Prospective, multi-center, randomized, blinded-endpoint (PROBE) trial	1 year	35 (aged 18–75 years)	Essential hypertension, albuminuria	Enalapril/lercanidipine 20/10 mg vs. enalapril/amlodipine 20/5 mg	Both combination treatments determined a similar reduction in BP. Combination therapy of enalapril/lercanidipine significantly reduced albuminuria.
Fici et al., 2020 [155]	Observational cross-sectional study	6 months	668	Essential hypertension, diabetes type I or II	Amlodipine/valsartan 5/160 mg vs. amlodipine/perindopril 10/5 mg vs. lercanidipine/enalapril 10/20 mg vs. verapamil/trandolapril 120/2 mg vs. nitrendipine/enalapril 10/20 mg vs. felodipine/ramipril 2.5/2.5 mg	The rate of reduction in albuminuria was the highest in the lercanidipine/enalapril group. There was no difference in the rate of BP reduction between the groups.

Abbreviations: ADR, adverse reactions; BP, blood pressure; DBP, diastolic blood pressure; SBP, systolic blood pressure.

## Data Availability

The data used in this article were sourced from materials mentioned in the References section.
